# Reactive Oxygen and Related Regulatory Factors Involved in Ethylene-Induced Petal Abscission in Roses

**DOI:** 10.3390/plants13131718

**Published:** 2024-06-21

**Authors:** Siwen Han, Jingjing Zhang, Wenyu Wang, Siying Zhang, Zhe Qin, Haixia Pei

**Affiliations:** School of Life Science and Technology, Inner Mongolia University of Science and Technology, Baotou 014010, China; 18247621907@163.com (S.H.); 15034732386@163.com (J.Z.); 15043814577@163.com (W.W.); 13170631964@163.com (S.Z.); qinzhe1990@outlook.com (Z.Q.)

**Keywords:** rose, ethylene, ROS, petal abscission, regulatory factors

## Abstract

Petal abscission affects the growth, development, and economic value of plants, but the mechanism of ethylene-ROS-induced petal abscission is not clear. Therefore, we treated roses with different treatments (MOCK, ETH, STS, and ETH + STS), and phenotypic characteristics of petal abscission, changed ratio of fresh weight, morphology of cells in AZ and the expression of *RhSUC2* were analyzed. On this basis, we measured reactive oxygen species (ROS) content in petals and AZ cells of roses, and analyzed the expression levels of some genes related to ROS production and ROS scavenging. Ethylene promoted the petal abscission of rose through decreasing the fresh weight of the flower, promoting the stacking and stratification of AZ cells, and repressing the expression of *RhSUC2*. During this process, ethylene induced the ROS accumulation of AZ cells and petals mainly through increasing the expressions of some genes (*RhRHS17*, *RhIDH1*, *RhIDH-III*, *RhERS*, *RhPBL32*, *RhFRS5*, *RhRAC5*, *RhRBOHD*, *RhRBOHC*, and *RhPLATZ9*) related to ROS production and repressing those genes (*RhCCR4*, *RhUBC30*, *RhSOD1*, *RhAPX6.1*, and *RhCATA*) related to ROS scavenging. In summary, ROS and related regulatory factors involved in ethylene induced petal abscission in roses.

## 1. Introduction

Abscission is a highly programmed process by which plant organs or tissues are separated from the main body, and it is the result of the separation of several layers of small and dense cell clusters in the Abscission Zone (AZ). The timely abscission of plant organs helps in the recycling and reusing of minerals in the plant body, maintaining balance between source and sink, promoting flowers’ pollination, and facilitating seed propagation, which is a successful strategy for plants to adapt to their living environment, and for survival and the continuation of species. However, for crops, the premature or abnormal abscission of economic organs caused by various factors seriously affects their yield and economic value.

Plant hormones are key factors in initiating abscission [1]. Ethylene, in particular, can induce the abscission of plants, while inhibitors of ethylene biosynthesis or signal transduction have opposite functions in ethylene-sensitive plants [2,3,4]. Most cells of AZ are sensitive to ethylene, although sensitivities are different [5,6]. In Arabidopsis (*Arabidopsis thaliana*), the flower organ shedding of ethylene insensitive mutants *ein2* and *etr1-1* is significantly delayed [7]. In tomato (*Solanum lycopersicum*), ethylene receptor mutants never river (*nr*), *sletr1-1*, and *sletr1-2* delay fruit ripening and abscission [8,9]. In rose (*Rosa hybrida*), the expressions of some key genes in ethylene biosynthesis and signal transduction (*ACS*, *ACO*, *ETRs*, *CTR*, *EIN2, EIN3/EIL*, and *ERFs*) are strongly induced by ethylene during rose petal senescence and abscission [10,11,12]. During the abscission process of rose petals, the ethylene content significantly increases, inducing the high expressions of *RbEXPA1* (α-expansion protein) and *RbXTH1/2* (the xylan glycosidase/hydrolase) [13,14]. In addition, *RhERF1* and *RhERF4* mediate ethylene- and auxin-regulated petal abscission by influencing pectin degradation [15].

Reactive oxygen species (ROS), serving as metabolic byproducts and secondary messengers, play a crucial role in growth, development, and environmentally triggered programmed cell death in plants [16,17]. ROS mainly includes singlet oxygen (^1^O_2_), superoxide (O_2_^−^), hydrogen peroxide (H_2_O_2_), and hydroxyl radicals (OH^−^), which have more active chemical activity than O_2_ [18]. Whether ROS is a second messenger or toxic substance mainly depends on the concentration [19]. The intracellular level of ROS is determined by the intricate interplay between ROS-producing pathways and ROS-scavenging mechanisms, which constitute a crucial part of the ROS gene network in plants [20]. Some genes that may affect ROS scavenging include superoxide dismutase (SOD), catalase (CAT), ascorbate peroxidase (APX), and glutathione peroxidase (GPX) [21]. As an antioxidant enzyme, SOD plays an important role in plant antioxidant stress [22], and it is involved in the reduction process of O_2_^−^ to H_2_O_2_ [23]. In addition, APX, CATn and GPX can act as scavengers to clear H_2_O_2_. APX is an ascorbic-acid-specific class I peroxidase that can act as a scavenger for hydrogen peroxide and as a sensor for changes in the redox state within plant cells, promoting ROS detoxification through the AsA-GSH cycle. CAT is a ubiquitous tetrameric heme-containing enzyme that catalyzes the dismutation of two molecules of H_2_O_2_ into H_2_O and O_2_ in a two-step reaction. GPX is a heme-free POX antioxidant enzyme that uses reduced glutathione (GSH) and thioredoxins (TRXs) to clear H_2_O_2_ [18].

Interestingly, ethylene has some physiological functions through affecting ROS. In regulating the response to salinity stress, ethylene largely functions by maintaining the homeostasis of Na^+^/K^+^, nutrients, and ROS [24]. In apple (*Malus pumila*), the shedding of young fruits along the cortical cells may be jointly regulated by the interaction among ABA, ethylene, and ROS [25,26]. In chili peppers (*Capsicum annuum*), H_2_O_2_ contributes to ethylene-induced leaf abscission [27]. However, in olives (*Canarium album*), ROS only responds to ethylene-induced leaf abscission, and not in the fruit [28]. In summary, the interaction between ethylene and ROS plays an important role in regulating plant organ abscission, but the molecular mechanism is complex and unclear.

Rose (*Rosa hybrida*) is one of the most important ornamental flowers worldwide. The abscission of rose petals, as an important factor in harvest loss, seriously affects the ornamental quality and economic value. However, understanding of the molecular mechanism of ethylene-ROS governing rose petal abscission is scarce. To this end, here, we found that ethylene induced the accumulation of ROS in rose petal and AZ during petal abscission, while STS (silver thiosulphate), an ethylene signaling inhibitor, played the opposite role. Our data indicated that the expressions of these genes (*RhRHS17*, LOC112172931; *RhIDH1*, LOC112172665; *RhIDH-III*, LOC112188192; *RhERS*, LOC112174439; *RhPBL32*, LOC112174003; *RhFRS5*, LOC112170513; *RhRAC5*, LOC112173840; *RhRAC1*, LOC112172302; *RhRBOHD*, LOC112196847; *RhRBOHC*, LOC112179306; *RhPLATZ9*, LOC112179143; *RhWRKY33a*, LOC112192343) involved in ROS production and those genes (*RhNOT9a*, LOC112167368; *RhCCR4*, LOC112200363; *RhUBC30*, LOC112185232; *RhSOD1*, LOC112200469; *RhAPX6.1*, LOC112172770; *RhAPX11.3*, LOC112173456; *RhCATA*, LOC112176552; *RhGPX1*, LOC112169965) involved in ROS scavenging were regulated by ethylene and STS. These genes may play important roles in regulating ethylene-induced petal abscission through the balance of ROS accumulation.

## 2. Materials and Methods

### 2.1. Plant Materials and Treatments

In this study, the ethylene-sensitive rose cultivar ‘Tineke’ (*R. hybrida* cv.) was used as the material for the experiments. Fresh rose flowers at stage 2 were harvested from a greenhouse in Baotou, Inner Mongolia Autonomous Region, China [15]. The roses were immediately taken back to the laboratory, trimmed to 25 cm in length, and placed in deionized water to rehydrate for 1–2 h at around 18 °C. The rose flowers were inserted into four kinds of treatment solutions containing, respectively, deionized water (MOCK), ETH (ethephon), STS (silver thiosulphate), and STS + ETH, with 36 flowers in each treatment, and the process was repeated at least three times. The STS solution was a mixed solution of AgNO_3_ (158 mg/L) and anhydrous Na_2_S_2_O_3_ (924 mg/L); the ETH solution was composed of 25 mg/L ethephon; and the mixed solution of STS and ETH was a 1:1 mixture of STS and ETH solutions. After the roses had been treated with the four solutions for 8 h, they were all inserted into solutions containing 8-Hydroxyquinoline (8-HQ) with a concentration of 200 mg/L, and this point was recorded as the starting point of day 1. Here, 8-HQ only acts as a bacteriostat [29]. The 8-HQ solution was replaced every day and the rose branches were cut a little under water daily. The roses in different treatments were placed in a culture room with a temperature of 23 ± 1 °C, relative humidity of 60~80%, and a 16/8 h (light/dark) photoperiod.

### 2.2. Observation of Abscission Phenotype in Rose Petal

Photographs were taken from the top angle of the rose flowers every 24 h using a Canon EOS 6D Mark II camera(Canon (China) Ltd., Beijing, China) to document the phenotypic changes during the abscission process. Observations continued until the petals had completely abscised or had dried up on the stems.

In addition, the change in the fresh weight of roses was determined. Each rose was weighed using an electronic balance (model PK2242H, manufactured by Aohaus Instruments, Changzhou, Co., Ltd., Changzhou, China) before and after pruning the roses every day. The first weight of the rose with the same length was recorded as W1, and the weight one day later was recorded as W2; the changed ratio of fresh weight (%) = (W2 − W1)/W1 × 100.

### 2.3. Collection of Abscission Zone Samples from Roses

The sampling standard for the abscission zone (AZ) is to cut the base of the petal and the receptacle where the petal is located less than 2 mm in length [4,30]. The collected AZ samples should be immediately frozen in liquid nitrogen and stored at −80 °C for subsequent qRT-PCR analysis.

### 2.4. Observation of the Cells in AZ of Rose Petal by Scanning Electron Microscopy

The AZ tissues were fixed in a 5% glutaraldehyde solution and then rinsed with phosphate buffer (0.1 M, pH 7.0). Subsequently, they were dehydrated in ethanol with concentrations of 30%, 50%, 70%, 80%, and 90%. The samples were treated with a mixed solution of ethanol and isoamyl acetate (*v*/*v* = 1/1) for 30 min, then treated with isoamyl acetate for 1 h. After critical drying and coating completion, the sample was observed using scanning electron microscopy (SEM, Phenom Pure desktop SEM, Eindhoven, The Netherlands).

### 2.5. Detection of ROS Contents

DCFH-DA (2′,7′-dichlorofluorescein diacetate) staining was performed to visually investigate the content of H_2_O_2_ in the AZ of rose. The tissues of AZ were incubated in 50 μM DCFH-DA solution at room temperature. After 30 min, the excess reagent was washed off and fluorescence images of the samples were obtained by laser confocal scanning microscope (Nikon Imaging Instruments Sales Co., Ltd. Shanghai, China) [31].

DAB (3′,3′-Diaminobenzidine) staining was used to visually investigate the content of H_2_O_2_ in rose petal. Four petals on the outer layer of one rose flower were selected, with five flowers as one group, and this was repeated at least 3 times. The petals were soaked in a DAB solution with a concentration of 1 mg/mL, and vacuum-extracted at 0.8 M pa for 5 min, and stained in the dark at 22 °C for 12 h. After the petals were rinsed with deionized water, they were decolorized overnight in a solution (volume ratio of glycerol/acetic acid/ethanol was 1:1:3). Subsequently, photos were taken of the samples and observed.

NBT (nitro blue tetazolium) staining was used to visually investigate the content of O_2_^−^ in rose petals. Samples were selected as above. The petals were soaked in an NBT solution with a concentration of 0.1 mg/mL. Vacuum suction and petal decolorization were performed as described above. The final samples were photographed for observation.

### 2.6. Quantifying the ROS Content

Image Pro Plus (IPP) 6.0 software (Media Cvbernetics, Rockville, MD, USA) was used to quantify ROS content in rose petals. (1) Set measurement parameters after importing images of DAB and NBT staining as follows: click the “Irregular AOI” option to manually outline the entire petal object, and click the “Count/Size” option to select the “Manual” option; then, click “Measure” to select “Select Measurements” and set “Per Area (Obj./Total)” as the measurement indicator, which can calculate the percentage of stained area relative to the total area. (2) Select and measure the stained area as follows: select the stained area of the rose petal within the AOI using “color picker”, and then click “Count” and the software will automatically calculate the area and provide the percentage relative to the total area based on the selected measurement indicator “Per Area (Obj./Total)”. (3) Analyze and collect data as follows: click “Statistics” and then select “View” to directly view the results or export the data to a file for further analysis.

Image Pro Plus (IPP) 6.0 software was also used to quantify the content of H_2_O_2_ in AZ of rose. (1) Set measurement parameters after importing images of DCFH-DA staining by clicking “Measure” to select “Calibration”, and “Intensity” as the measurement indicator, which can calculate fluorescence intensity in the selected area, and then click “Count/Size” to select “Measure”; then, click “Select Measurements” to select “Area”. (2) Select and measure the fluorescence intensity area by clicking “Count/Size” to select “Manual”, and click “Select Colors” to select “Histogram Based”; then, select the fluorescence area and click “Count”. The software will automatically calculate the fluorescence intensity of the area. (3) Analyze and collect the data as above.

### 2.7. RNA Extraction and RT-qPCR

The samples of AZ were collected for qRT-PCR validation in rose. The extraction of total RNA was carried out according to the kit instructions (pBIOZOL Plant Total RNA extraction Kit, Beijing Bomase Technology Development Co., LTD., Beijing, China). Add the ground powder (<1 g) to a 1.5 mL RNase free centrifuge tube, and then add 0.5 mL of pBIOZOL pre-cooled at 4 °C, and mix well. Let it stand at room temperature for 5–10 min, and centrifuge with 12,000 rpm for 2 min, and then transfer the supernatant to a new RNase free centrifuge tube. Add 100 μL NaCl (5 M) and mix well. Then, add 300 μL chloroform and mix well. Transfer the upper aqueous phase to a new RNase-free centrifuge tube, and then centrifuge with 12,000 rpm for 10 min at 4 °C. Add isopropanol at the same volume as the previous liquid, mix well, and let it stand at 4 °C for 10 min, and then centrifuge with 12,000 rpm for 10 min at 4 °C. Add 1 mL 75% ethanol to the tube after removing the supernatant, and then centrifuge with 12,000 rpm for 5 min. Discard the liquid, and allow the remaining ethanol to air-dry naturally. Dissolve RNA in an appropriate amount of RNase-free water. RNA bands were detected using agarose gel electrophoresis, and RNA concentration was measured by Nano Drop 2000 UV spectrophotometer (Nanodrop Company, Wilmington, DE, USA).

Reverse transcription was performed using an Evo M-MLV RT Mix Kit with gDNA Clean (Accurate Biology, Changsha, China). QRT-PCR was performed using SYBR Green Pro HS qPCR kit (Accurate Biology, Changsha, China) and a 7500 Fast Real-Time PCR System (Applied Biosystems, Foster City, CA, USA). The reaction system was set as follows: 1 µL cDNA as template, 0.8 µL (10 μmol·L^−1^) per primer, 10 µL 2×YBR Green *Pro Taq* HS Premix, 0.4 µL ROX Reference Dye (10 μmol·L^−1^), and 2 µL RNase free water. The reaction procedure was set as follows: 95 °C for 30 s, 60 °C for 5 s, 95 °C for 34 s, 60 °C for 15 s, 95 °C for 1 min, and 60 °C for 15 s with 40 cycles. *RhUBI2* (Gene ID: LOC112181994) was used as the internal reference gene [30], and all the sequences of the gene primers were shown in Supplementary Table S1. The primers were designed using the NCBI online tool. The expression levels were calculated by 2^−ΔΔCT^.

### 2.8. Statistical Analysis and Image Processing

ANOVA analysis of variance was performed using SPSS (version 24.0, Chicago, IL, USA), and graphs were plotted using Origin 2018 (Microcal Software, Northampton, MA, USA) and Prism, version 8.0 (GraphPad Software Inc., San Diego, CA, USA). Image processing was performed using Photoshop CC 2019 (Adobe, San Jose, CA, USA) software.

## 3. Results

### 3.1. Ethylene Promotes Petal Abscission by Affecting Cells of AZ and Related Physiological Indicators

**Figure 1:**
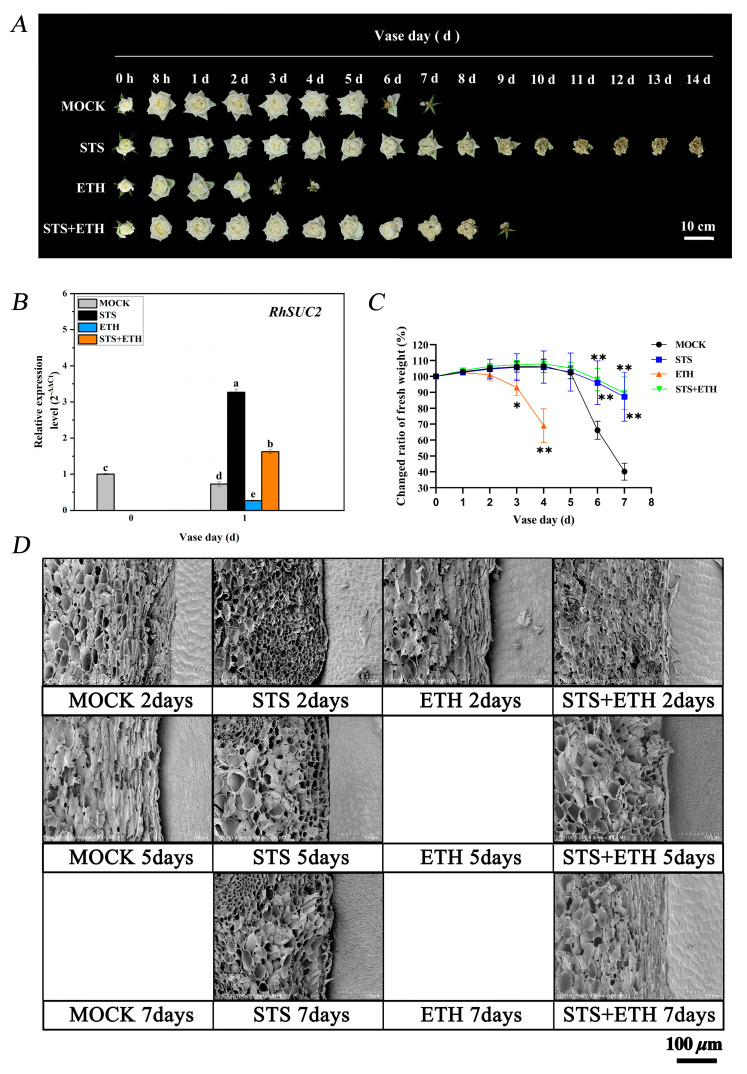
Phenotype and related physiological indicators. (**A**) The phenotypic characteristics of rose petal abscission under different treatments: 0 h, no treatment; 8 h, 8 h of treatment with different solutions; 1 to 14 days, days after treatment; scale bars 10 cm. (**B**) The expression of sucrose transporter (*RhSUC2*) in rose AZ. Values are mean ± SD of three biological replicates. Bars with different letters are significantly different at *p* < 0.05 according to Duncan’s multiple range tests. (**C**) Changed rate of fresh weight in rose. Statistical significance between different treatment and control groups was tested using Duncan’s test (* *p* < 0.05; ** *p* < 0.01); biological replicates n = 3. (**D**) Morphological observations of AZ cells in rose. Scale bars = 100 μm.

To evaluate the potential role of ethylene and STS in the petal abscission process of roses, phenotypic characteristics of petal abscission, the changed ratio of fresh weight, the morphology of cells in AZ and the expression of sucrose transporter *RhSUC2* were analyzed. It is shown in Figure 1A that the time order of petal abscission was ETH > MOCK > STS + ETH > STS. The rose petals abscised completely with a treatment of deionized water (MOCK) after about 7 days, and these petals treated with ETH abscised completely after about 4 days, while the petals treated with STS did not abscise, and were wilted or withered by the end of the experiment. Petals with a treatment of STS + ETH abscised completely after about 9 days (Figure 1A).

Previous studies have shown that a low expression of *RhSUC2* can lead to petal abscission earlier in rose [30]; here, we used *RhSUC2* as a marker gene for the timing of petal abscission. After 1 day of treatment, compared with the MOCK, the expression level of *RhSUC2* significantly decreased by 0.6 times in ETH treatment, significantly increased by 3.5 times in STS treatment, and significantly increased by 1.2 times with STS + ETH treatment (Figure 1B).

A decrease in fresh weight is usually one of the characteristics of flower abscission and senescence [32]. Therefore, we calculated the changed ratio of fresh weight under different treatments in rose. In the MOCK group, the rose showed strong water absorption capacity from day 1 to day 3, with a gradual increase in the changed ratio of fresh weight. However, on day 4, the trend reversed and showed a decline. In the ETH group, the changed ratio of fresh weight began to decline from the second day, accompanied by the start of petal abscission, and maintained a downward trend until the petals abscised completely on day 4. In the STS and STS + ETH groups, the changed ratio of fresh weight of the roses showed similar trends, with a gradual increase from day 0 to 4. From the fifth day, the changed ratio of fresh weight began to slowly decrease, but remained at a relatively high level compared to the MOCK and ETH groups (Figure 1C).

To better observe the AZ structure of roses under different treatments, we collected the AZ tissue at the connection site between petals and receptacles and observed the cell morphology through scanning electron microscopy. On day 2 in the ETH group, the AZ cells were loosely arranged and formed a large number of vesicular cells, while the cells at the junction were accumulated and layered. Similar phenomena occurred on the fifth day in MOCK, and on day 7 in STS + ETH. However, until the seventh day, no obvious similar phenomenon was observed in the STS group (Figure 1D).

The above results indicate that ethylene induced the abscission of rose petals by decreasing the expression of *RhSUC2*, inhibiting the water absorption of petals and promoting morphological changes in AZ cells, while STS played an opposite role in this process.

### 3.2. Ethylene Enhanced the Accumulation of H_2_O_2_ and O_2_^−^ in Rose Petals

**Figure 2:**
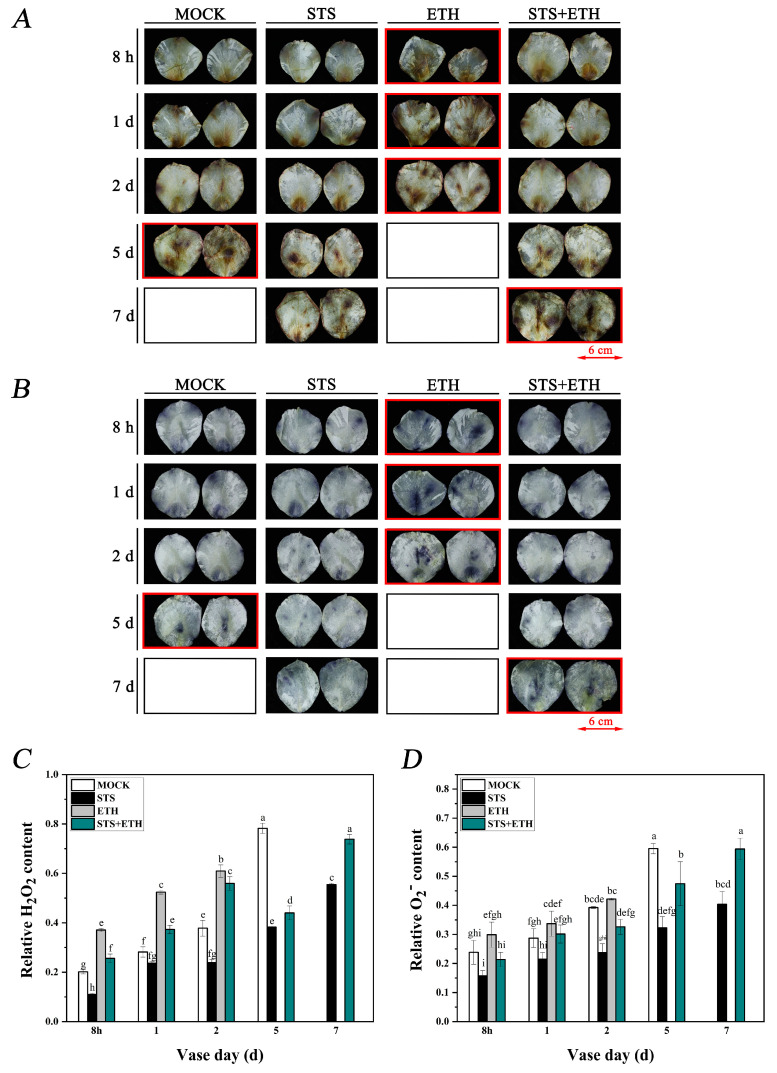
The content of H_2_O_2_ and O_2_^−^ in rose petals. (**A**) DAB staining of rose petals treated with MOCK, ETH, STS, and STS + ETH. Scale bars = 6 cm. (**B**) NBT staining of rose petals treated with MOCK, ETH, STS, and STS + ETH. Scale bars = 6 cm. (**C**) H_2_O_2_ content was indicated by a quantitative analysis of DAB staining with Image Pro Plus (IPP) 6.0 software. (**D**) O_2_^–^ content was indicated by a quantitative analysis of NBT staining with Image Pro Plus (IPP) 6.0 software. The red boxes indicate significant deepening of the dyeing color or significant increase in area. Values are mean ± SD of three biological replicates. Bars with different letters are significantly different at *p* < 0.05 according to Duncan’s multiple range tests.

As one of the earliest cellular responses during abscission and senescence, ROS production has been reported to play a crucial role in regulating this process [20,33,34]. The rose petals stained with DAB formed brown spots, indicating the accumulation of H_2_O_2_. From 8 h to the second day, there were deeper brown and larger brown spots in petals treated with ethylene. Subsequently, a quantitative analysis was conducted on the DAB staining through Image Pro Plus (IPP) 6.0 software; the quantitative values of the brown spots in the ETH group were significantly higher than those in other groups, suggesting that ethylene promoted the production of H_2_O_2_. On day 5, a large number of brown spots appeared on the petals in the MOCK group, while only a small amount of brown spot in the STS and STS + ETH groups. The quantitative values in STS and STS + ETH groups were significantly lower than that in MOCK group. STS maintained a low level of H_2_O_2_ even on day 7, indicating that STS inhibited the accumulation of H_2_O_2_ in rose petals during the abscission process (Figure 2A,C). Rose petals stained with NBT form blue pots, indicating the accumulation of O_2_^−^. The staining results of NBT were similar to those of the DAB staining. Ethylene also enhanced the production of O_2_^−^, while STS inhibited the accumulation of O_2_^−^ in rose petals during the abscission process (Figure 2B,D).

### 3.3. Ethylene Promoted H_2_O_2_ Production in the AZ of Rose Petals

**Figure 3:**
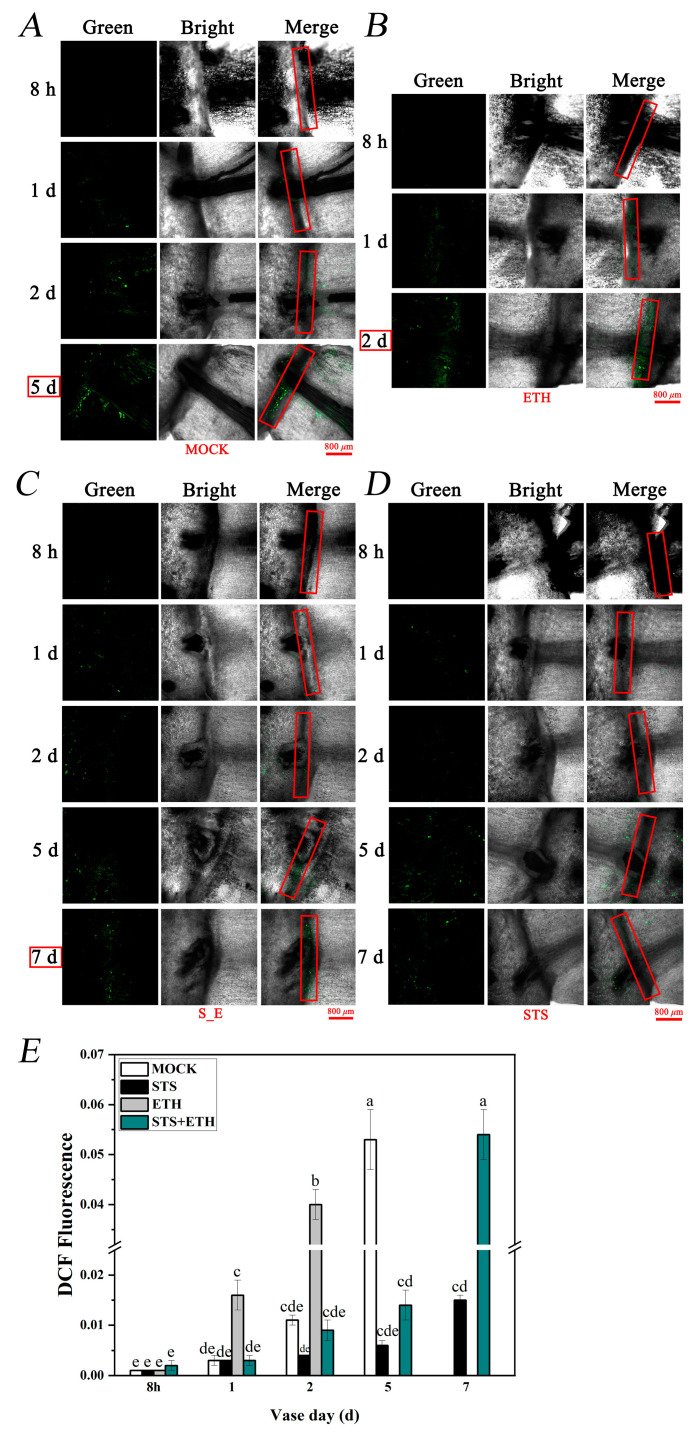
H_2_O_2_ level in the AZ of rose petal. (**A**–**D**) DCFH-DA staining and observation of AZ treated with MOCK, ETH, STS, and STS + ETH in rose petals. Scale bar = 800 μm. (**E**) H_2_O_2_ content was indicated by a quantitative analysis of intensity of green fluorescence in AZ with Image Pro Plus (IPP) 6.0 software. The red boxes indicate AZ of rose petals. Bars with different letters are significantly different at *p* < 0.05 according to Duncan’s multiple range tests.

AZ is the direct site where the petals abscise from the mother body, so we focused on whether ethylene and STS also affect ROS generation in AZ during the abscission process of rose petals. The petal AZ was stained with the fluorescent indicator dye 2,7-dichlorodihydrofluorescein diacetate (DCFH-DA), which produced green fluorescence, indicating the accumulation of H_2_O_2_ [35]. On day 2, banded green fluorescence was observed only in the AZ of petals treated with ethylene, indicating that ethylene increases the H_2_O_2_ content in AZ during petal abscission (Figure 3B). On day 5, stronger green fluorescence was observed in the MOCK group, while only weaker green fluorescence was produced in the STS and STS + ETH groups (Figure 3A,C,D). On day 7, significantly stronger green banded fluorescence was exhibited in the STS + ETH group compared to the STS group. Subsequently, a quantitative analysis was conducted on the fluorescence results (Figure 3E). The results showed that ethylene significantly increased the H_2_O_2_ level, while STS significantly inhibited the H_2_O_2_ level. This suggested that ethylene may induce the occurrence of abscission by increasing the H_2_O_2_ content in the AZ of rose petals, while STS plays the opposite role in this process.

### 3.4. Ethylene Regulated the Expressions of Key Genes Related to ROS Production and ROS Scavenging in the AZ of Rose Petals

**Figure 4:**
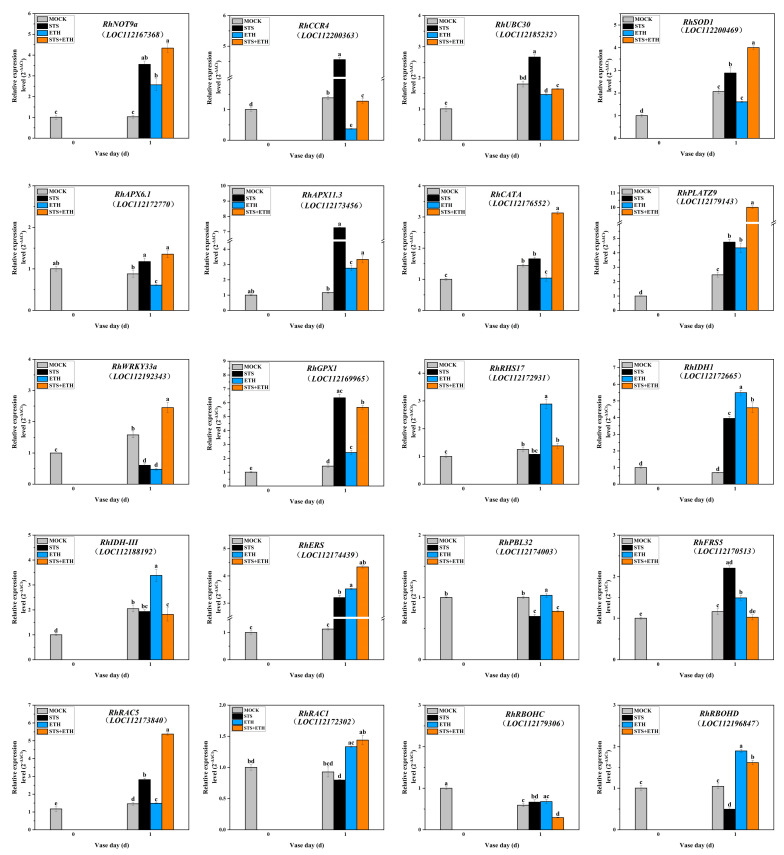
Relative expression levels of key genes related to ROS production (*RhRHS17*, *RhIDH1*, *RhIDH-III*, *RhERS*, *RhPBL32*, *RhFRS5*, *RhRAC5*, *RhRAC1*, *RhRBOHD*, *RhRBOHC*, *RhPLATZ9*, and *RhWRKY33a*) and ROS scavenging (*RhNOT9a*, *RhCCR4*, *RhUBC30*, *RhSOD1*, *RhAPX6.1*, *RhAPX11.3*, *RhCATA*, and *RhGPX1*) in AZ of rose petals. Values are mean ± SD of three biological replicates. Bars with different letters are significantly different at *p* < 0.05 according to Duncan’s multiple range tests.

The accumulation of ROS depends on the balance between its production and scavenging. We found that ethylene induced petal abscission in rose, and ROS was significantly increased in this process. Based on this, we speculate that ethylene may affect the expressions of related genes involved in the production and scavenging of ROS. According to an in-depth analysis of our previous transcriptome data, the expressions of some genes that may affect ROS levels were screened and detected (Figure 4).

We have focused on some genes that may affect ROS scavenging. *RhNOT9a* and *RhCCR4*, encoding superoxide dismutase, constitute CCR4-NOT transcription complex subunit 9. Compared with MOCK, ethylene only significantly decreased the expression of *RhCCR4*, while the expressions of *RhNOT9a* and *RhCCR4* were significantly increased by STS. Compared with MOCK, the expression levels of *RhUBC30* and *RhSOD1* encoding two superoxide dismutases were significantly downregulated by ETH and upregulated by STS. The expression levels of the key genes (*RhAPX6.1* and *RhAPX11.3*) encoding ascorbate peroxidase were detected. *RhAPX6.1* was significantly downregulated by ETH and upregulated by STS, and *RhAPX11.3* was significantly upregulated by ETH and STS, although the increased extent under ETH treatment was comparatively smaller compared to STS. The expression level of a key gene, *RhCATA*, encoding catalase was significantly downregulated by ETH and upregulated by STS. *RhGPX1*, a key gene encoding glutathione peroxidase, was significantly upregulated by ETH and STS, although the upregulation extent under ETH treatment was comparatively smaller compared to STS. The above results indicate that the expression levels of some ROS-related scavenging genes were significantly repressed by ethylene and induced by STS.

In addition, we also detected the expressions of some genes that may be involved in the generation of ROS. The RhWRKY33a-RhPLATZ9-RhRbohD module maintains redox homeostasis as a brake [32]. Therefore, the expression levels of *RhPLATZ9*, *RhWRKY33a*, and *RhRbohD* were also measured. Compared to MOCK, the expression of *RhPLATZ9* gene was significantly upregulated under ETH, STS, and STS + ETH treatments. The expression of *RhWRKY33a* gene was significantly downregulated under ETH and STS treatments, and was significantly upregulated under STS + ETH treatment. *RhRBOHD* was significantly upregulated by ETH and significantly downregulated by STS. Meanwhile, another burst oxidase homolog protein, *RhRBOHC*, was also detected. However, the expression level of *RhRBOHC* did not exhibit significant change under ETH or STS treatment. The expression levels of the key genes encoding isocitrate dehydrogenase, *RhRHS17* (root hair specific 17), *RhIDH1* and *RhIDH-III* (isocitrate dehydrogenase 1 and III), *RhERS* (glutamate-tRNA ligase), *RhPBL32* (PBS1-Like 32), and *RhFRS5* (FAR1-related sequence 5) exhibited different patterns under STS and ETH treatments compared to MOCK. ETH significantly increased expressions of *RhRHS17*, *RhIDH1*, *RhIDH-III*, *RhERS*, *RhPBL32*, and *RhFRS5*, while STS decreased the expression levels of *RhRHS17*, *RhIDH-III*, and *RhPBL32*, but significantly increased the expressions of *RhERS* and *RhFRS5*.

Interestingly, the expression level of *RhRAC1* (rac-like GTP-binding protein 1), which has dual roles as an inducer of ROS production and an inhibitor of ROS scavenging [36], was upregulated by ETH, while not significantly inhibited by STS. In addition, the expression level of *RhRAC5*, another rac-like GTP-binding protein, was significantly downregulated by ETH, and upregulated by STS.

These results indicate that ETH and STS had complex roles in modulating ROS production and scavenging in the petal abscission of rose.

## 4. Discussion

**Figure 5:**
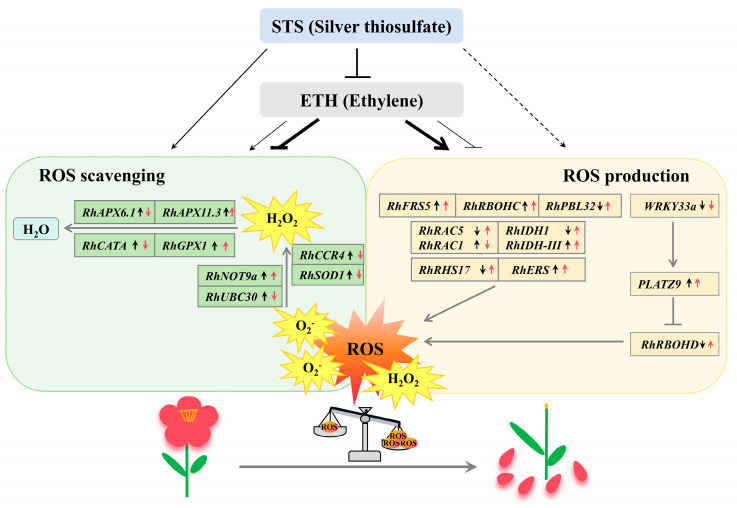
Model of ethylene-induced petal abscission in rose by regulating the expression of ROS-related genes. Ethylene and STS regulated the expressions of genes in ROS-production and ROS-scavenging, respectively. The red arrow behind the gene represents the result regulated by ethylene, and the black one represents the result regulated by STS. The thickness of the lines below the ethylene box indicates that ethylene mainly induces the expressions of ethylene generation genes and inhibits the expressions of ethylene scavenging genes, but the opposite result also exists (thin lines). The independent arrow under the STS box indicates that in addition to its role as an ethylene inhibitor, STS may also promote the expressions of these genes in other ways.

Plant organ shedding is necessary for plant development and survival [37]. Here, we reported that ethylene may affect the accumulation of ROS by regulating the expression of ROS generation and scavenging related genes, thereby inducing petal abscission in rose (Figure 5).

It is well known that ethylene usually promotes the shedding of plant organs, but the specific regulatory mechanism is not entirely clear. Here, the ethylene-sensitive rose cultivar ‘Tineke’ was used as the research material. STS is a known inhibitor in ethylene [15], so it was also applied in this study to better understand the function of ethylene in the petal abscission of rose. We observed the phenotypes of roses under ethylene treatment from the outside to the inside. At the individual level, ethylene significantly accelerated the abscission of rose petals, which was consistent with the previously reported results of ethylene promoting plant organ shedding [4,15,38,39] (Figure 1A). At the physiological level, ethylene rapidly reduced the fresh weight of rose flowers (Figure 1C). Similar phenomena have also been reported in other studies; ethylene effectively participates in Zinc oxide nanoparticle (ZnO NP)-induced toxicity and ultrastructural and stomatal damage in rice (*Oryza sativa*) seedlings, and significantly reduces the fresh weight of rice shoots and roots [40]. At the cellular level, the cells of AZ were rapidly promoted to stack and stratify by ethylene in rose petals; in addition, the AZ cells were loosely arranged and formed a large number of vesicular cells treated by ethylene (Figure 1D). In tomato, there have been similar results, and the overexpression of the ethylene response transcription factor *ERF116* made AZ cells in plants stay in a partially detached state, with larger and less-staining cell gaps and a looser arrangement [41]. If *RhSUC2* (sucrose transporter) is expressed at a low level, it will induce the abscission of rose petals [30]. At the molecular level, we used *RhSUC2* as a marker gene for an indicator about the timing of petal abscission, and its expression was significantly reduced by ethylene (Figure 1B). Taken together, these results demonstrate that ethylene plays a positive role in the regulation of petal abscission in rose.

Usually, ROS can induce plant organ abscission, while ROS scavengers inhibit plant organ abscission [27]. However, the role of ROS in different plant organ shedding processes is not entirely the same. For example, in olives (*Canarium album*), ROS only responds to ethylene-induced leaf abscission, and not in the fruit [28]. Currently, the regulatory mechanism of ROS on rose petal abscission is not clear. We found that ethylene induced the accumulation of ROS both in rose petals and in AZ cells of rose petals according to DAB, NBT, and DCFH-DA staining, while the effect of STS was opposite to this (Figure 2 and Figure 3). Although this result is almost as expected, we have obtained firm experimental data on ethylene-induced ROS in the abscission of rose petals. Moreover, it is worth exploring in future research whether a small amount of ROS has a positive effect on delaying petal abscission, while a large amount of ROS promotes petal abscission.

The level of ROS determines whether it is harmful or beneficial in plant growth, development, and stress response, while the accumulation of ROS depends on the balance between its production and scavenging. The genes that regulate the production and clearance of ROS are not exactly the same in different plants and processes. We wonder which genes are involved in the balance regulation of ROS during the process of ethylene-induced rose petal abscission. All of four SOD genes, *RhNOT9a*, *RhCCR4*, *RhUBC30*, and *RhSOD1*, were significantly down-regulated and up-regulated by ETH and STS, respectively, except for *RhNOT9a*. The overexpression of the *OsCu*/*ZnSOD* gene in rice reduced salt-induced oxidative damage by improving ROS’s detoxification ability [23]. These results suggest that ETH can inhibit the expression of some SOD genes, weaken the detoxification of ROS, and aggravate the oxidative damage. The expression level of gene *RhNOT9a* increased under ETH treatment, which may be in order to resist the ethylene effect and maintain the balance of ROS in plants. In addition, CAT, APX, and GPX can act as scavengers to clear H_2_O_2_. Some genes encode these enzymes, such as *RhCATA*, *RhAPX6.1*, *RhAPX11.3*, and *RhGPX1*, and their expression levels were also significantly downregulated and upregulated by ETH and STS. The above results indicate that ETH perhaps accelerates the accumulation of ROS by reducing the expressions of genes related to ROS scavenging, while STS can reduce oxidative damage through upregulating genes related to ROS scavenging. However, there are also ROS scavenging genes that are upregulated by ETH, such as *RhAPX11.3* and *GPX1*. We speculated that these two genes may be involved in ethylene-induced ROS accumulation through antagonistic regulation or other more complex forms in the petal abscission of rose.

The expressions of some genes that may be involved in the generation of ROS were also detected. As expected, genes including *RhRHS17*, *RhIDH1*, *RhIDH-III*, *RhERS*, *RhPBL32* and *RhFRS5* that positively regulate ROS generation were upregulated by ETH, while STS decreased the expression levels of *RhRHS17*, *RhIDH-III*, and *RhPBL32* among them. Interestingly, the expressions of *RhERS* (glutamate-tRNA ligase) and *RhFRS5* (FAR1-related sequence 5) were also upregulated by STS, and this may be involved in oxidative stress caused by STS containing silver ions. Studies have confirmed that silver nanoparticles (AgNPs) can significantly induce excessive ROS production in marine diatom Skeletonema costatum [42]. Here, *RhERS* and *RhFRS5* may be more sensitive to silver ions compared to STS as an ethylene inhibitor.

Previous studies have shown that the RhWRKY33a-RhPLATZ9 regulatory module inhibits the rapid accumulation of ROS in rose (*Rosa hybrida* cv. Samantha). ROS-induced *RhWRKY33a* directly upregulates *RhPLATZ9* expression, and *RhPLATZ9* can downregulate *RhRBOHD* expression to prevent ROS accumulation [31]. In our study, the *RhWRKY33a* gene was downregulated by both ETH and STS, and this showed that *RhWRKY33a* may participate in both ethylene-induced ROS accumulation and oxidative stress caused by STS containing silver ions. Inexplicably, the expression of the *RhPLATZ9* gene was upregulated by ETH, STS, and STS + ETH. We seem to be unable to provide a reasonable explanation for this result as yet, and can only speculate that perhaps its regulation to ROS also has duality. However, *RhRBOHD* was significantly increased by ETH and significantly decreased by STS, and another burst oxidase homolog protein, *RhRBOHC*, did not exhibit significant change under ETH or STS treatment. This indicated that although they both belong to the same category of burst oxidase homolog protein, their functions are not entirely the same. However, in Arabidopsis, Ca^2+^ can activate *RBOHC* and *RBOHD*-dependent ROS production; at this time, their functions are the same [43,44].

Interestingly, the expression of *RhRAC1* (rac-like GTP-binding protein 1) can be increased by ETH, while not being inhibited by STS in our result. In rice, *OsRac1* plays a dual role as an inducer of ROS production and a suppressor of ROS scavenging [44]. Our result showed *RhRAC1* can be an inducer of ROS production during the ethylene-induced petal abscission of rose, and STS can act as an inhibitor of ethylene, but it not only has this function, but also has other functions [4,45,46,47,48]. In summary, ethylene and STS regulated the expressions of related genes related to ROS production and scavenging in a complex manner to induce ROS accumulation in the petal abscission of rose.

## 5. Conclusions

Our results demonstrate that ethylene promoted the petal abscission of rose through decreasing the fresh weight of flowers, promoting the stacking and stratification of AZ cells, and repressing the expression of *RhSUC2* (sucrose transporter). During this process, ethylene induced the ROS accumulation of AZ cells and petals, mainly through increasing the expressions of some genes (*RhRHS17*, *RhIDH1*, *RhIDH-III*, *RhERS*, *RhPBL32*, *RhFRS5*, *RhRAC5*, *RhRBOHD*, *RhRBOHC*, and *RhPLATZ9*) related to ROS production and repressing those genes (*RhCCR4*, *RhUBC30*, *RhSOD1*, *RhAPX6.1*, and *RhCATA*) related to ROS scavenging. In a word, reactive oxygen and related regulatory factors involved in ethylene induced petal abscission in roses (Figure 5).

## Data Availability

Data will be made available on request.

## Supplementary Materials

The following supporting information can be downloaded at: https://www.mdpi.com/article/10.3390/plants13131718/s1, Supplementary Table S1: Sequences of the primers.
